# Effects of Autologous Platelet-Rich Plasma on Healing of Peptic Ulcers: A Randomized Controlled Trial

**DOI:** 10.1155/2022/7944849

**Published:** 2022-07-15

**Authors:** Ting Xu, Yin Tian, Yi Wang, Zhongmei Yi, Chenchen Li, Shichun Wang, Yahan Fan, Chunyan Yao, Guiyong Peng, Hua Lu

**Affiliations:** ^1^Department of Blood Transfusion, Southwest Hospital, Third Military Medical University (Army Medical University), Chongqing, China; ^2^Department of Gastroenterology, Southwest Hospital, Third Military Medical University (Army Medical University), Chongqing, China; ^3^Department of Rehabilitation Medicine, Southwest Hospital, Third Military Medical University (Army Medical University), Chongqing, China; ^4^Department of Blood Transfusion, The Second Affiliated Hospital of Chongqing Medical University, Chongqing, China

## Abstract

**Purpose:**

Peptic ulcer is a multifactorial and complex disease and affects a wide range of people worldwide. We provided a novel therapeutic approach for peptic ulcer and observed its effect.

**Methods:**

Peptic ulcer patients were enrolled from 2016 to 2017 in Chongqing and randomly assigned to two groups: a control group that used only rabeprazole and a platelet-rich plasma (PRP) group that received a combination therapy of autologous PRP (aPRP) and rabeprazole. The therapeutic effect was assessed via the ulcer size and symptom score.

**Results:**

A total of 27 patients were included (12 patients in the control group and 15 patients in the PRP group) in this study. Our results showed that all participants have healed in 30 days, and there was no significant difference in healing time between the PRP group and the control group in different independent variables. However, regression analysis revealed that the healing time was 6.99 days shorter in the PRP group than that in the control group, and patients with higher symptom scores in the initial examination need more time to heal during treatment. Endoscopic results showed that the repaired ulcer in the PRP group was more similar to the normal gastric mucosa tissue than that the control group.

**Conclusion:**

This study showed an encouraging preliminary result that aPRP has a positive result in patients with peptic ulcer and seems to be a better choice for refractory peptic ulcer treatment. Although further follow-up studies are needed to determine the duration of efficacy of aPRP, the approach will be helpful in improving the clinical treatment of peptic ulcer.

## 1. Introduction

Peptic ulcer is a common digestive disease in the modern society and included gastric and duodenal ulcer [1]. The lifetime prevalence of peptic ulcer was 5-10% of the worldwide population, and its incidence rate ranged from 0.1% to 0.3% per year [2, 3]. In some regions of China, the prevalence rate of endoscopically confirmed peptic ulcer reached 17.2% in the general population, and most patients with peptic ulcer were asymptomatic [4]. However, the exact pathogenesis of peptic ulcer remains unclear [5]. Recently, researchers have demonstrated that peptic ulcer is caused by a combination of multiple factors, such as excessive secretion of gastric acid and *Helicobacter pylori* (*H. pylori*) infection [6–8]. Therefore, the conventional therapy of peptic ulcer is a combination treatment through the use of antibacterial, antacids, and protective mucosa drugs. However, due to the lack of active repair measures of gastric mucosa injury, the treatment time is relatively long, and the adverse effects are often inevitable [9].

Autologous platelet-rich plasma (aPRP) was derived from autologous blood and contains high concentrations of platelets, which can secrete a large number of cell growth factors through the release of intracellular *α*-granules after activation [10, 11]. These growth factors can stimulate the proliferation of fibroblasts, endothelial cells, and other cells, to help enhance the active repair and anti-infective ability of the wounded tissue [12, 13]. Additionally, many studies have reported that aPRP is a simple and low-cost therapeutic option in clinical practice and has been successfully applied to some conditions, such as bone regeneration, aesthetic procedures, muscle and tendon repair, refractory wound, and diabetic ulcer [14, 15].

Hence, we proposed a combined treatment option to treat peptic ulcers with the positive effects of aPRP and drugs, in terms of shortening the treatment time and improving the quality of ulcers healing, reducing the adverse effects of drugs, and increasing the velocity of complete ulcers healing. We present our preliminary observations in this paper.

## 2. Materials and Methods

### 2.1. Ethical Statements

This study has been registered in the Chinese Clinical Trial Registry (http://www.chictr.org.cn), registered in 14/12/2015, and the registration number was ChiCTR-ONN-15007573. This clinical trial was approved by the Ethics Committee of Southwest Hospital, Third Military Medical University (Army Medical University), Chongqing, China (number: 2015 Scientific Research No. (63)). All methods were performed in accordance with the Declaration of Helsinki. We obtained the written informed consent from all participants before the initiation of the trial. Participants' names were replaced by codes in this study, and all medical record information was kept strictly confidential to protect the privacy rights of participants.

### 2.2. Participants

**Figure 1:**
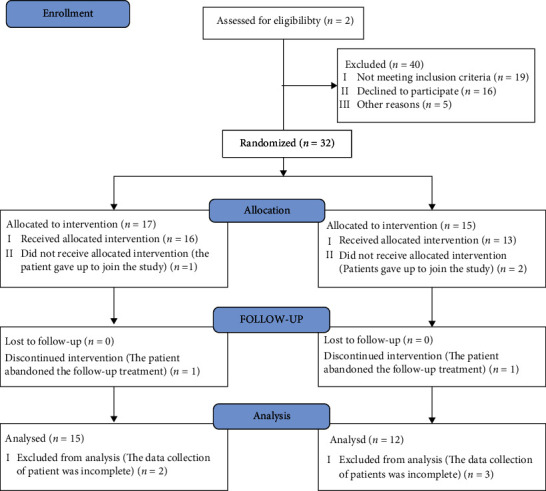
Procedure for participating in this study. A total of 32 patients were included in this study from March 1, 2016, to July 31, 2018, and 3 patients did not receive allocated intervention because of the absence of therapy, and 2 patients discontinued intervention for their personal reasons.

A total of 32 patients were included in this study from March 2016 and July 2017. All participants are volunteers and the demographic details of participants are shown in Table 1. Among the participants, 33.33% were female and 66.67% were male, and 59.25% were aged 41-60 years. The recruitment and treatment of participants were completed in Southwest Hospital. The diagnosis of peptic ulcer was based on the digestive system endoscopy. If peptic ulcer patients were found to meet the enrolled criteria and agreed to participate in this study, they were assigned into the platelet-rich plasma (PRP) and control groups according to the randomization sequence (random number table method). The symptom score of patients varied from 3 to 11. Of 27 patients, 15 were assigned into the PRP group and received the combination therapy of aPRP and rabeprazole, and 12 patients were assigned into the control group and received drug therapy. The process of enrollment is presented in Figure 1.

Patients were included in this trial if they meet the following criteria:

- Diagnosis of peptic ulcer confirmed by digestive system endoscopy and diameter of ulcer ≥ 5 mm
- Age between 18 and 60 years
- Absence of hematopoietic disease and cardiopulmonary insufficiency
- Blood platelet count ≥ 100 × 10^9^/L
- Negative test results for human immunodeficiency virus (HIV), hepatitis B virus (HBV), hepatitis C virus (HCV), and syphilis
- Absence of pregnancy
- Participants were not used antiplatelets or nonsteroidal anti-inflammatory drugs (NSAIDs)

### 2.3. Preexperiment of Platelet Aggregation Function after Acid Treatment In Vitro

Preliminary experiments in vitro were carried to detect the function of platelets in the acidic environment similar to the stomach. The counts of apheresis platelets obtained were adjusted to 200-300 × 10^9^/L and then divided into the treatment group and control group. 112.5 *μ*L PRP of the treatment group were treated with pH 2.5 hydrochloric acid (HCl) for 30 s, and 112.5 *μ*L PRP of control group were treated with 112.5 L normal saline for 30 s. And then platelet aggregation was detected by a platelet aggregator (Manufacturer: Helena; Model: AggRAM; Manufacturing location: USA), activated with ADP (final concentration 1.25 *μ*M) and epinephrine (final concentration 18.75 *μ*M).

### 2.4. Autologous PRP Preparation

Initially, 100 mL of blood was collected from participants' antecubital vein and stored in the blood preservation bags (Nigale Co. Ltd., Chengdu, People's Republic of China) that contain trisodium citrate. The blood was centrifuged for 10 min at 1,800 × *g* in 22°C. 25 mL of buffy coat (supernatant) was transferred to the new bag, and 75 mL of erythrocytes (precipitation) was discarded after centrifugation. Then, the buffy coat was centrifuged for 15 min at 200× *g* in 22°C. After centrifugation, 15 mL of PRP (supernatant) was transferred to the new bag and the erythrocytes (precipitation) were discarded. Finally, the PRP was oscillated for 30 min in 22°C and then stored at -80°C after the platelet count (leucocytes were removed in this preparation process, so the percentage of leucocytes was not specifically mentioned). The platelet concentration in aPRP was fourfold to sixfold higher than the initial concentration, which was considered a qualified aPRP, and it is not activated before application (the activation process of aPRP is completed by some related factors in vivo). Additionally, the aggregation rate of aPRP at pH 2.5 and 7.0 (the concentration of measured PRP was adjusted to 200-300 × 10^9^/L) was measured via an aggregation remote analyzer module (Helena Laboratories Co. Ltd., Texas, USA) after being activated via the addition of adrenaline and adenosine diphosphate (final concentration of 18.75 *μ*mol/L and 1.25 *μ*mol/L, respectively).

### 2.5. Interventions

All patients enrolled in this study were randomized into the PRP and control groups. Participants were not allowed to use antiplatelets or nonsteroidal anti-inflammatory drugs during the therapy course of the study. In the control group, patients received drug therapy, and the drug used was rabeprazole (Livzon Co. Ltd., Zhuhai, People's Republic of China), which is a new type of proton pump inhibitors and widely used in therapy for digestive system diseases (note: due to the rabeprazole being more effective than lansoprazole, we used rabeprazole in the experiment, which was approved by the ethics committee). The dose of the drug was 20 mg/day, and it should not be continuously used for >45 days. In the PRP group, patients received a combination therapy of aPRP and drug (the drug used was the same as that in the control group), and the treatment should last <45 days. In aPRP therapy, aPRP was used to coat the peptic ulcer surface of patients via the gastroscopy forceps tube (generally, a 1 cm ulcer needs to be coated with 5 mL PRP). Ulcers healing status was observed in all patients through gastroscopy every 10 days. If the ulcer has healed, the patients were evaluated for therapeutic effect, and the therapy was terminated. If the ulcer has not healed, the patients continued to undergo treatment until the deadline. The therapy was performed no more than five times, and the time interval between treatments was 10 days. The final results were assessed at therapy termination or the deadline.

### 2.6. Measured Parameters

We collected the patient's personal information and clinical characteristics, including age, sex, ulcer type, ulcer size (diameter), and symptom severity (we acquired the sample size by the statistical power calculation, and the result was 80%). Among them, ulcer type and size were determined via gastroscopy. The symptom severity of the ulcer was determined using the total symptom score, which was the sum of the four symptom scores that include burning sensation, epigastric pain, acid reflux, and bloating, and each of these was on a scale of 0 (normal) to 3 (serious) [16]. In this assessment, the highest total score was 12 points, and the more severe clinical symptoms were indicated in the higher score.

Therapeutic effect evaluation was interpreted as follows:

- Healing: the ulcer has healed, the symptom score was 0, and the clinical symptoms have disappeared.
- Effective: the ulcer size and symptom score have decreased by 60%, and the clinical symptoms are improved to some degree.
- Noneffective: the ulcer size and symptom score have not decreased, and the clinical symptoms are not changed or worse.

### 2.7. Statistical Analysis

Statistical analysis was performed using IBM SPSS 20.0 (SPSS Co. Ltd., Chicago, USA). Quantitative variables were calculated as mean and standard deviation. Independent-sample *t*-test or chi-square test was used to compare the difference in each variable. Regression analysis was used to assess the effect of multiple factors on ulcer healing (27 patients were included in the regression analysis). The independent variables included age, gender, ulcer type, ulcer size, symptom score, group, and enrollment time. The 95% confidence intervals (CI) were calculated for all associations.

## 3. Results

### 3.1. Participants

A total of 32 patients were included in this study from March 1, 2016, to July 31, 2018, and 5 patients were excluded because of the absence of therapy (Table 1). Among the participants, 33.33% were female and 66.67% were male, and 59.25% were aged 41-60 years. The peptic ulcer type in most patients was duodenal ulcer. Additionally, the ulcer size in all patients were >5 mm, and 10 patients in this study had ulcer size > 8 mm. The symptom score of patients varied from 3 to 11 of 27 patients, 15 were assigned into the PRP group and received the combination therapy of aPRP and rabeprazole, and 12 patients were assigned into the control group and received drug therapy.

### 3.2. The Result of Preexperiment of Platelet Aggregation Function after Acid Treatment In Vitro

**Figure 2:**
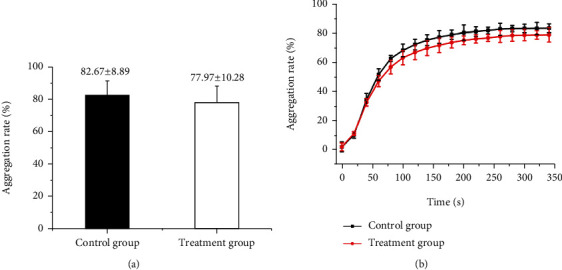
Comparison of aggregation rate between the treatment group and control group. (a) Comparison of aggregation rate between the treatment group (*n* = 30) and control group (*n* = 30). The aggregation rate of the treatment group was lower without any significant difference to that of the control group (77.97 vs. 82.67%, *P* > 0.05). (b) Comparison of time point between the treatment group (*n* = 30) and control group (*n* = 30); aPRP reached the maximum aggregation rate. The time point of the treatment group and the control group was 240 and 260 s, respectively (*P* > 0.05).

Our results showed that the aggregation rate in the treatment group (treated with pH 2.5 HCl) was lower than that in the control group, but there is no significant difference (77.97 vs. 82.67%, *P* > 0.05, Figure 2(a)). Additionally, similar results were obtained for the difference in the time point that aPRP reached the maximum aggregation rate in the control and treatment groups (260 s vs. 240 s, *P* > 0.05, Figure 2(b)). Therefore, we considered that the biological function of aPRP in the gastrointestinal tract is not significantly attenuated within a certain amount of time. Moreover, the pH of the internal environment of the stomach can rise above 5 by acid suppressants, so it is feasible to use aPRP in the treatment of peptic ulcer disease.

### 3.3. Comparison of Therapeutic Efficacy between the PRP and Control Groups

As shown in Table 2, in the PRP and control groups, the mean ulcer size in the initial examination was 6.73 and 6.25 mm, respectively, and the mean symptom score before the intervention was 5.86 and 5.83, respectively. However, there was no significant difference in ulcer size and symptom score between the two groups (*P* > 0.05). Additionally, there was also no significant difference in healing time (17.33 days vs. 21.67 days, *P* > 0.05).

All patients of this study experienced healing in 30 days (Table 3). In the PRP group, 5 patients experienced healing in 10 days with PRP therapy only once, and 9 patients experienced healing in 20 days with PRP therapy twice. In the control group, 1 patient experienced healing in 10 days after drug therapy, and 8 patients experienced healing in 20 days after drug therapy. However, there was no significant difference in the therapeutic efficacy at the same therapeutic intervals in the two groups (*P* > 0.05).

We analyzed and compared the patient's healing time in different variables (Table 4). In both men and women, our results showed that there was no significant difference in ulcer size, symptom score, and healing time between the two groups (*P* > 0.05). In patients aged 18-40 years, the ulcer size and symptom score were smaller and lower, respectively, in the PRP group than those in the control group (*P* > 0.05), and the mean healing time in the PRP group was shorter but without any significant difference to that of the control group (17.14 days vs. 17.50 days, *P* > 0.05). In those aged 41-60 years, the PRP group had significantly larger ulcer size than the control group (*P* < 0.01), but there was no significant difference in healing time between the two groups (*P* > 0.05). A total of 15 patients were enrolled in the first half of the year, and the PRP and control groups had no significant difference in ulcer size and symptom score (*P* > 0.05). However, the healing time was significantly shorter in the PRP group than that in the control group (15.00 days vs. 22.86 days, *P* < 0.05). Additionally, 12 patients were enrolled in the second half of the year, and the mean healing time in the PRP group was similar to that in the control group with no significant difference (20.00 days vs. 20.00 days, *P* > 0.05).

Additionally, we used the regression model to evaluate the effect of multiple factors on the healing time of ulcer (Table 5). The independent variables included age, sex, ulcer type, ulcer size, symptom score, group, and enrollment time. The results showed that the healing time in the PRP group was 6.99 days shorter than that in the control group (*P* < 0.01; 95% CI, 2.15-11.84), and patients with higher symptom scores in the initial examination need more time to heal in therapy (*P* < 0.05; 95% CI, 0.34-2.62).

**Figure 3:**
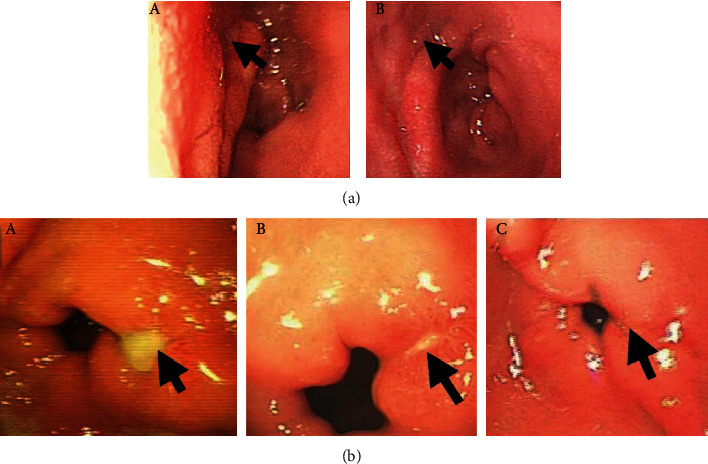
Observation for aPRP therapy for peptic ulcer. (a) A 46-year-old female patient had an 8 mm duodenal ulcer and received the combination therapy of aPRP and rabeprazole. (A) Before the treatment. (B) After 10-day treatment. (b) A 35-year-old male patient had an 8 mm gastric ulcer and was cured only by rabeprazole. (A) Before the treatment. (B) After 10-day treatment. (C) After 20-day treatment. The arrow shows the ulcer. It can be seen that the gastric mucosa in the PRP group is closer to the normal gastric mucosa after treatment, while the scar tissue filling in the control group is more obvious.

Finally, we found that the repaired ulcer in PRP group was more similar to the normal gastric mucosa tissue than that the control group by endoscopic (Figure 3(a)), while the control group had more scar tissue filling and obvious contracture (Figure 3(b)).

## 4. Discussion

In this study, we provided a novel therapeutic approach for patients with peptic ulcer through a combination therapy of aPRP and rabeprazole. Compared to the conventional therapy, this was a breakthrough treatment for promoting ulcers healing in which aPRP was applied on the ulcer surface via a gastroscopy pipeline. Additionally, aPRP treatment has some advantages for patients with refractory peptic ulcer compared to the long-term drug therapy, such as short time and few side effects. Our results showed that aPRP therapy has the ability to accelerate the healing process in peptic ulcers, and the healing time was 6.99 days shorter in the PRP group than in the control group, without any recurrent and serious adverse events in the enrolled patients in the 6-month follow-up. Additionally, we have not received any side effects reports from the control group.

Peptic ulcer and its complications are still a challenge due to its high mortality and death rates [17]. A higher incidence rate is usually noted in people who smoke, consume alcohol, or use NSAIDs [18, 19]. However, 316 million individuals are current smokers in China, and 68.6% of males and 42.6% of females consumed alcohol [20, 21]. The high prevalence rate of smoking and alcohol consumption may be the reasons that the incidence rate of peptic ulcer is higher in China than in Western countries.

Several drugs are presently available for the prevention and treatment of peptic ulcer in clinical practice. H2-receptor antagonists and proton pump inhibitors are commonly used in peptic ulcer therapy, but all have adverse effects. Previous studies have reported that H2-receptor antagonists cause headache, pancreatitis, and confusion in elderly patients, and proton pump inhibitors also cause some side effects, such as itching, skin rash, diarrhea, and dizziness [22]. Additionally, a combination therapy of a proton pump inhibitor and two antibiotics has been used to treat *H. pylori* infection in gastrointestinal ulcers to eradicate this pathogen [18]. This therapeutic approach can relieve the symptoms of peptic ulcer and improve the eradication rate of *H. pylori* infection, but several researchers have reported that this therapy is not universally effective and causes serious problems in the treatment of peptic ulcer [9]. The most important thing is that the above treatments are only for the cause of gastric mucosal injury (gastric acid microorganisms), but for the gastric mucosa that has been damaged, they can only wait for its natural healing with scar tissue filling to varying degrees, but not the active proliferation of original tissue. So the course of treatment is relatively long, and it is not the best choice for the recovery of gastric mucosa function.

Ulcer healing requires mucosal proliferation. Numerous studies suggested that PRP offers a high level of growth factors to enhance the healing of wounded tissues, and the idea of using aPRP in the treatment of peptic ulcers originated from these studies [23–25]. Additionally, previous studies have proved that PRP therapy was no significant safety risks in different settings. Arslan et al. have demonstrated that aPRP injection appears to be an effective treatment and might lead to positive results in the vision of patients with retinitis pigmentosa and have not observed any serious adverse events in patients in the 1-year follow-up [26]. Yol et al. investigated the therapeutic efficacy of PRP on tissue maturation and wound healing in experimental colonic anastomosis, and their results showed that PRP may improve the anastomotic strength of patients with impaired wound healing in colon anastomosis [27].

Both heterologous and autologous PRP can release a large number of growth factors to stimulate cell proliferation and then enhance the repair of wounded tissues. However, there is still a risk of allergic reactions and infectious disease transmission in heterologous PRP [28]. We preferred to use aPRP in our study to avoid these problems because aPRP was prepared from autologous blood and a sterile operation was needed in the whole process. Additionally, it is necessary in our study that the PRP has the capability of aggregation in treated areas because these growth factors were released after platelet aggregation [29]. However, most studies have shown that PRP is commonly used in surgical and open wounds, and it has not been reported to be used in peptic ulcers. Thus, we have concerns that the biological function of PRP is affected by gastric acid in the gastrointestinal tract. Fortunately, our results showed that PRP function does not seem to be affected at pH 2.5.

Moreover, we found that a reasonable dietary pattern has a positive effect on ulcers healing in this study. The research center site was located in Chongqing, and all patients come from this area. Chongqing is an inland city in Southwest China, and the weather is mostly cloudy with rain and humidity. As a result of the weather condition, local residents like to add some seasoning on the food, such as chili peppers and Chinese prickly ash, and spicy food can affect gastrointestinal function and further cause gastrointestinal disease. This may be an important reason that the incidence rate of peptic ulcer is higher in Chongqing than that in other regions and why many patients with peptic ulcer in this region have more severe conditions and are more difficult to treat in the long term [30]. Thus, we strictly required that all patients should not eat spicy food as much as possible during the course of the treatment, and the enrolled patients fully complied with this requirement according to the follow-up results. In this trial, all patients have healed in 30 days, and the healing time was shorter than the common therapy time (4-6 weeks). It was indicated that a reasonable dietary pattern may also play an important role in peptic ulcer treatment, which can help promote ulcer healing.

Finally, we reported a novel effective approach for peptic ulcer treatment, but this approach has several limitations. On the one hand, patients were required to have good physical status because aPRP was obtained from autologous blood and patients may also have to overcome the negative emotion caused by blood donation and frequent gastroscopy. Therefore, the number of participants was much lower than expected, and this may also be the reason that our studies have no significant difference in different independent variables. However, we calculated the power regarding the number of patients necessary for this study. The results showed that when 1-*β* was 80% and *α* was 0.05, the required sample size for each group was 11 cases. So, we believe that the existing sample size can comply with the requirements of this study. However, we will continue to increase the number of participants to obtain more clinical trial data in further research. On the other hand, peptic ulcer is a recurrent disease, and the follow-up period was only 6 months. It is not clear how long aPRP effects will last, and the effects and duration may vary from patient to patient. Although we have not received any reports of recurrence or side effects during this period, extended follow-up time is important to help us for acquiring the exact time of aPRP effect. This was considered another important limitation of this study and should be addressed in future research. Finally, we found that aPRP repaired ulcers mostly through the proliferation of the original tissue, which may be more conducive to maintaining the normal function of gastric mucosa. However, further pathological and functional studies are needed to confirm this result.

## Figures and Tables

**Table 1:** The personal information and clinical characteristics of patients.

No.	Age	Gender	Ulcer type	Diameter of ulcer (mm)^a^	Symptom score^b^	Group	Enrollment time (month/day/year)	Healing time (days)^c^
1	37	Male	Gastric ulcer	5	4	PRP	03/30/2016	10
2	25	Male	Duodenal ulcer	6	4	PRP	06/17/2016	10
3	43	Male	Duodenal ulcer	8	6	PRP	06/20/2016	20
4	46	Female	Duodenal ulcer	8	9	PRP	06/21/2016	10
5	52	Male	Duodenal ulcer	5	4	PRP	07/04/2016	10
6	37	Female	Duodenal ulcer	5	4	PRP	09/05/2016	20
7	49	Female	Duodenal ulcer	5	7	Control	09/22/2016	30
8	35	Male	Gastric ulcer	8	6	PRP	11/02/2016	20
9	44	Female	Duodenal ulcer	5	7	Control	11/07/2016	20
10	47	Male	Duodenal ulcer	8	11	PRP	11/17/2016	20
11	37	Male	Duodenal ulcer	6	5	PRP	11/23/2016	20
12	44	Male	Duodenal ulcer	8	8	PRP	11/29/2016	30
13	50	Male	Duodenal ulcer	6	3	Control	12/15/2016	20
14	52	Male	Duodenal ulcer	5	5	Control	02/14/2017	20
15	43	Male	Duodenal ulcer	7	5	PRP	03/08/2017	10
16	30	Male	Duodenal ulcer	6	5	PRP	03/15/2017	20
17	48	Male	Duodenal ulcer	8	6	PRP	03/23/2017	20
18	46	Male	Duodenal ulcer	6	8	Control	04/18/2017	20
19	54	Female	Duodenal ulcer	5	6	Control	04/19/2017	30
20	20	Female	Duodenal ulcer	7	5	Control	04/25/2017	20
21	35	Male	Duodenal ulcer	5	5	PRP	05/18/2017	20
22	32	Female	Duodenal ulcer	6	6	Control	05/27/2017	20
23	50	Male	Duodenal ulcer	8	8	Control	06/02/2017	30
24	22	Female	Duodenal ulcer	8	8	Control	06/08/2017	20
25	53	Male	Duodenal ulcer	8	6	PRP	07/03/2017	20
26	51	Male	Duodenal ulcer	6	3	Control	07/03/2017	20
27	28	Female	Duodenal ulcer	8	4	Control	07/21/2017	10

No.: the enrollment number of patients; PRP: platelet-rich plasma. ^a^The diameter of ulcer was measured before the intervention. ^b^The score of clinical symptom evaluation in the initial examination. ^c^Because the ulcer healing status was observed in all patients through gastroscopy every 10 days, the actual healing time may be shorter than that shown in this table.

**Table 2:** Comparison of healing time between the PRP group and control group in peptic ulcer healing.

Category	No.	Diameter of ulcer^a^	Symptom score^b^	Healing time (d)
PRP group	15	6.73 ± 1.33	5.86 ± 2.03	17.33 ± 5.94
Control group	12	6.25 ± 1.22	5.83 ± 1.85	21.67 ± 5.77
*P* value^c^		>0.05	>0.05	>0.05

No.: number of patients; PRP: platelet-rich plasma. ^a^The size of ulcer was measured before the intervention. ^b^The score of clinical symptom was evaluated in the initial examination. ^c^Independent sample *t* test was used to test statistical difference in the distribution with each group.

**Table 3:** Comparison of therapeutic efficacy at different therapeutic intervals in peptic ulcer healing.

Group	Number of patients	Number of healing patients		
		10 d^a^	20 d	30 d
PRP	15	5 (33.33%)^b^	9 (60.00%)	1 (6.67%)
Control	12	1 (8.33%)	8 (66.67%)	3 (25.00%)
*P* value^c^		>0.05	>0.05	>0.05

PRP: platelet-rich plasma. ^a^Healing time (days). ^b^The number of healing patients at this interval has a percentage of the total number of patients in this group. ^c^Chi-square trend test was used to test statistical difference in the distribution with each group.

**Table 4:** Comparison of healing time in different variables in peptic ulcer healing.

Variables	Group	No.	Ulcer size (mm)^a^	Symptom score^b^	Healing time (d)
Gender					
Male	PRP	13	6.77 ± 1.30	5.77 ± 1.92	17.69 ± 5.99
	Control	5	6.20 ± 1.10	5.40 ± 2.51	22.00 ± 4.47
*P* value^c^			>0.05	>0.05	>0.05
Female	PRP	2	6.50 ± 2.12	6.50 ± 3.54	15.00 ± 7.07
	Control	7	6.29 ± 1.38	6.14 ± 1.35	21.43 ± 6.90
*P* value^c^			>0.05	>0.05	>0.05
Age					
18-40	PRP	7	5.86 ± 1.07	4.71 ± 0.76	17.14 ± 4.88
	Control	4	7.25 ± 0.96	5.75 ± 1.71	17.50 ± 5.00
*P* value^c^			>0.05	>0.05	>0.05
41-60	PRP	8	7.50 ± 1.07	6.88 ± 2.30	17.50 ± 7.07
	Control	8	5.75 ± 1.04	5.88 ± 2.03	23.75 ± 5.18
*P* value^c^			<0.01	>0.05	>0.05
Enrollment time					
First half year	PRP	8	6.63 ± 1.30	5.50 ± 1.60	15.00 ± 5.34
	Control	7	6.43 ± 1.27	6.57 ± 1.40	22.86 ± 4.88
*P* value^c^			>0.05	>0.05	<0.05
Second half year	PRP	7	6.86 ± 1.46	6.28 ± 2.49	20.00 ± 5.77
	Control	5	6.00 ± 1.10	4.80 ± 1.83	20.00 ± 6.32
*P* value^c^			>0.05	>0.05	>0.05

No.: number of patients; PRP: platelet-rich plasma. ^a^The size of ulcer was measured before the intervention. ^b^The score of clinical symptom was evaluated in the initial examination. ^c^Independent sample *t* test was used to test statistical difference in the distribution with each group.

**Table 5:** Regression analysis between the different variables in peptic ulcer healing.

Variables	Regression coefficients	*P* value^c^	95% CI^b^
Group	6.99 ± 2.34	<0.01	2.15-11.84
Symptom score^a^	1.48 ± 0.55	<0.05	0.34-2.62

^a^The score of clinical symptom was evaluated in the initial examination. ^b^95% confidence interval. ^c^Chi-square trend test was used to test the statistical difference in each group.

## Data Availability

In order to protect the patients' privacy, the original data of this study were not disclosed.

## Abbreviations

PRP: Platelet-rich plasma
NSAIDs: Nonsteroidal anti-inflammatory drugs
*H. pylori*: *Helicobacter pylori*
aPRP: Autologous platelet-rich plasma
PLT: Platelet count
HIV: Human immunodeficiency virus
HBV: Hepatitis B virus
HCV: Hepatitis C virus
CI: Confidence intervals.

## References

[1] Périco L. L., Emílio-Silva M. T., Ohara R. (2020). Systematic analysis of monoterpenes: advances and challenges in the treatment of peptic ulcer diseases. *Biomolecules*.

[2] Lanas A., Chan F. K. (2017). Peptic ulcer disease. *Lancet*.

[3] Hooi J. K., Lai W. Y., Ng W. K. (2017). Global prevalence of *Helicobacter pylori* infection: systematic review and meta-analysis. *Gastroenterology*.

[4] Li Z., Zou D., Ma X. (2010). Epidemiology of peptic ulcer disease: endoscopic results of the systematic investigation of gastrointestinal disease in China. *The American Journal of Gastroenterology*.

[5] Datta D., Roychoidhury S. (2015). To be or not to be: the host genetic factor and beyond inHelicobacter pylorimediated gastro-duodenal diseases. *World Journal of Gastroenterology*.

[6] Sverdén E., Brusselaers N., Wahlin K., Lagergren J. (2018). Time latencies of _Helicobacter pylori_ eradication after peptic ulcer and risk of recurrent ulcer, ulcer adverse events, and gastric cancer: a population-based cohort study. *Gastrointestinal Endoscopy*.

[7] Sarri G. L., Grigg S. E., Yeomans N. D. (2019). Helicobacter pylori and low-dose aspirin ulcer risk: a meta-analysis. *Journal of Gastroenterology and Hepatology*.

[8] Molaoa S. Z. (2021). Prevalence ofHelicobacter pyloriinfection and the incidence of the associated malignant and peptic ulcer disease (PUD) at Nelson Mandela Academic Hospital: a retrospective analysis. *Journal of Drug Assessment*.

[9] Scally B., Emberson J. R., Spata E. (2018). Effects of gastroprotectant drugs for the prevention and treatment of peptic ulcer disease and its complications: a meta-analysis of randomised trials. *The Lancet Gastroenterology & Hepatology*.

[10] Schär M. O., Diaz-Romero J., Kohl S., Zumstein M. A., Nesic D. (2015). Platelet-rich concentrates differentially release growth factors and induce cell migration in vitro. *Clinical Orthopaedics and Related Research*.

[11] Eren G., Gürkan A., Atmaca H., Dönmez A., Atilla G. (2016). Effect of centrifugation time on growth factor and MMP release of an experimental platelet-rich fibrin-type product. *Platelets*.

[12] Sharara F. I., Lelea L. L., Rahman S., Klebanoff J. S., Moawad G. N. (2021). A narrative review of platelet-rich plasma (PRP) in reproductive medicine. *Journal of Assisted Reproduction and Genetics*.

[13] Peng G. L. (2019). Platelet-rich plasma for skin rejuvenation: facts, fiction, and pearls for practice. *Facial Plastic Surgery Clinics of North America*.

[14] Everts P., Onishi K., Jayaram P., Lana J. F., Mautner K. (2020). Platelet-rich plasma: new performance understandings and therapeutic considerations in 2020. *International Journal of Molecular Sciences*.

[15] Everts P. A., van Erp A., DeSimone A., Cohen D. S., Gardner R. D. (2021). Platelet rich plasma in orthopedic surgical medicine. *Platelets*.

[16] Bo T. J. (2018). Efficacy of rabeprazole in the treatment of active gastric ulcer and ulcer healing analysis in adult. *Journal of Clinical Medical*.

[17] Tarasconi A., Coccolini F., Biffl W. L. (2020). Perforated and bleeding peptic ulcer: WSES guidelines. *World Journal of Emergency Surgery : WJES*.

[18] Kavitt R. T., Lipowska A. M., Anyane-Yeboa A., Gralnek I. M. (2019). Diagnosis and treatment of peptic ulcer disease. *The American Journal of Medicine*.

[19] Yegen B. C. (2018). Lifestyle and peptic ulcer disease. *Current Pharmaceutical Design*.

[20] Li S. S., Ma C. W., Xi B. (2016). Tobacco control in China: still a long way to go. *Lancet*.

[21] World Health Organization (2018). *Country profile: China. In global status report on alcohol and health 2018*.

[22] Strand D. S., Kim D., Peura D. A. (2017). 25 years of proton pump inhibitors: a comprehensive review. *Gut and Liver*.

[23] Anitua E., Muruzabal F., Tayebba A. (2015). Autologous serum and plasma rich in growth factors in ophthalmology: preclinical and clinical studies. *Acta Ophthalmologica*.

[24] Alsousou J., Thompson M., Harrison P., Willett K., Franklin S. (2015). Effect of platelet-rich plasma on healing tissues in acute ruptured Achilles tendon: a human immunohistochemistry study. *Lancet*.

[25] Yi Z. M., Jiang T. L., Xu X. (2017). Prestorage application of preoperative autologous platelet gel combined with whole blood in 1 case. *Chongqing Medicine*.

[26] Arslan U., Özmert E., Demirel S., Örnek F., Şermet F. (2018). Effects of subtenon-injected autologous platelet-rich plasma on visual functions in eyes with retinitis pigmentosa: preliminary clinical results. *Graefe's Archive for Clinical and Experimental Ophthalmology*.

[27] Yol S., Tekin A., Yilmaz H. (2008). Effects of platelet rich plasma on colonic anastomosis. *The Journal of Surgical Research*.

[28] Kieb M., Sander F., Prinz C. (2017). Platelet-rich plasma powder: a new preparation method for the standardization of growth factor concentrations. *The American Journal of Sports Medicine*.

[29] Amable P. R., Carias R. B., Teixeira M. V. T. (2013). Platelet-rich plasma preparation for regenerative medicine: optimization and quantification of cytokines and growth factors. *Stem Cell Research & Therapy*.

[30] Lu M., Sun G., Zhang X. M. (2018). Peptic ulcer is the most common cause of non-variceal upper-gastrointestinal bleeding (NVUGIB) in China. *Medical Science Monitor*.

